# Clinical Audit of Psychotropic Medication Use in People With Learning Disabilities and Behaviour That Challenges

**DOI:** 10.1192/bjo.2025.10649

**Published:** 2025-06-20

**Authors:** Johnson Olajolumo, Imelda Ogar

**Affiliations:** Tees, Esk and Wear Valley NHS Foundation Trust, Durham, United Kingdom

## Abstract

**Aims:** To evaluate compliance with best practices in prescribing psychotropic medications for individuals with learning disabilities and behaviours that challenge, in line with National Institute For Health and Care Excellence (NICE) guidelines and the Stopping Over-Medication of People with a Learning Disability, Autism, or Both initiative.

**Methods:** A retrospective audit was conducted on five patients prescribed psychotropic medications between January 2023 and December 2024 at the Chester-Le-Street Adult Learning Disability Community Team. Data were extracted from electronic patient records using a structured audit tool aligned with NICE NG11 standards.

**Results:** Strengths:

100% compliance in documenting the rationale for prescribing.

100% ensured psychotropic medication was used alongside psychological interventions.

100% identified comorbid conditions influencing behaviour.

Areas for Improvement:

Timely medication reviews: Only 20% had effectiveness and side effects reviewed within the recommended 3–4 weeks.

Treatment duration documentation: Absent in 100% of cases.

Patient/carer involvement: Considered in 40% of cases.

Multidisciplinary team (MDT) reviews: Completed within three months in only 40% of cases.

**Conclusion:** The audit demonstrates strong adherence to prescribing rationale and psychological intervention use but identifies significant gaps in medication monitoring, patient involvement, and MDT reviews. To enhance patient safety and adherence to national guidelines, the following recommendations are made:

1. Standardizing early medication reviews within 3–4 weeks.

2. Improving documentation of treatment duration.

3. Enhancing patient and carer engagement in medication decisions.

4. Ensuring timely MDT reviews to optimize prescribing practices.

Implementing these changes will support safer psychotropic prescribing, reduce unnecessary medication use, and promote a holistic approach to managing challenging behaviours in people with learning disabilities.

